# Parkinson’s disease is characterized by vitamin B6-dependent inflammatory kynurenine pathway dysfunction

**DOI:** 10.21203/rs.3.rs-4980210/v1

**Published:** 2024-09-26

**Authors:** Edward Wilson, Jacob Umans, Michelle Swarovski, Paras Minhas, Øivind Midttun, Arve Arve Ulvik, Marian Shahid-Besanti, Patricia Linortner, Siddhita Mhatre, Qian Wang, Divya Channappa, Nicole Corso, Lu Tian, Carolyn Fredericks, Geoffrey Kerchner, Edward Plowey, Brenna Cholerton, Per Ueland, Cyrus Zabetian, Nora Gray, Joseph Quinn, Thomas Montine, Sharon Sha, Frank Longo, David Wolk, Alice Chen-Plotkin, Victor Henderson, Tony Wyss-Coray, Anthony Wagner, Elizabeth Mormino, Nima Aghaeepour, Kathleen Poston, Katrin Andreasson

**Affiliations:** Stanford University; Stanford University; Stanford University; Stanford University; Bevital; Bevital; Stanford University; Stanford University; Stanford University; Stanford University; Stanford University; Stanford University; Stanford University; Stanford University; F. Hoffmann-La Roche, Ltd.; Stanford University; Stanford University; Bevital; VA Puget Sound Health Care System and University of Washington Seattle; Oregon Health & Sciences University; Oregon Health and Sciences University; Stanford University; Stanford University; Stanford University; University of Pennsylvania; Perelman School of Medicine at the University of Pennsylvania; Stanford University; Standford; Stanford University; Stanford University; Stanford University; Stanford University; Stanford University

**Keywords:** Parkinson’s disease, plasma, cerebrospinal fluid, Kynurenine pathway, vitamin B, inflammation, metabolism

## Abstract

Parkinson’s disease (PD) is a complex multisystem disorder clinically characterized by motor, non-motor, and premotor manifestations. Pathologically, PD involves neuronal loss in the substantia nigra, striatal dopamine deficiency, and accumulation of intracellular inclusions containing aggregates of α-synuclein.

Recent studies demonstrate that PD is associated with dysregulated metabolic flux through the kynurenine pathway (KP), in which tryptophan is converted to kynurenine (KYN), and KYN is subsequently metabolized to neuroactive compounds quinolinic acid (QA) and kynurenic acid (KA). This multicenter study used highly sensitive liquid chromatography-tandem mass-spectrometry to compare blood and cerebral spinal fluid (CSF) KP metabolites between 158 unimpaired older adults and 177 participants with PD. Results indicate that increased neuroexcitatory QA/KA ratio in both plasma and CSF of PD participants associated with peripheral and cerebral inflammation and vitamin B6 deficiency. Furthermore, increased QA tracked with CSF tau and severity of both motor and non-motor PD clinical dysfunction. Importantly, plasma and CSF kynurenine metabolites classified PD participants with a high degree of accuracy (AUC = 0.897). Finally, analysis of metabolite data revealed subgroups with distinct KP profiles, and these were subsequently found to display distinct PD clinical features. Together, these data further support the hypothesis that the KP serves as a site of brain and periphery crosstalk, integrating B-vitamin status, inflammation and metabolism to ultimately influence PD clinical manifestation.

## INTRODUCTION

Parkinson’s disease (PD) is a multisystem neurodegenerative condition characterized by a loss of dopaminergic neurons in the substantia nigra pars compacta and aggregation of misfolded α-synuclein protein in Lewy bodies across the brainstem and other structures^1^. Striatal dopamine depletion contributes to classical PD motor and non-motor clinical dysfunction, the latter of which may precede appearance of motor symptoms by years or decades. Furthermore, significant heterogeneity exists in the presentation of PD symptoms – which often fluctuate – and disease progression varies greatly between patients^2^. Given the unpredictable nature in the presentation and progression of PD, there is a pressing need to develop novel biomarkers capable of predicting PD pathophysiology and clinical presentation^3^.

Multiple lines of evidence implicate central nervous system (CNS) and peripheral inflammation in the PD initiation and progression^4–6^. Indeed, genetic studies have identified PD risk variants relating to immune function^7^. Postmortem examinations have demonstrated reactive microglia and incursion of immune cells in PD brain^8^. Moreover, imaging markers of neuroinflammation^9,10^ and biofluid biomarkers^11–14^ reveal chronic immune activation in PD *in vivo*. Despite these findings, the mechanisms linking inflammation and PD remain unclear.

The kynurenine pathway (KP) is modified by peripheral and CNS inflammation, and improper KP metabolism leads to the accumulation of proinflammatory and neuroactive intermediate metabolites, collectively termed kynurenines. Under physiological conditions, tryptophan (TRP) is converted to kynurenine (KYN), which is subsequently metabolized via B vitamin-dependent reactions to neuroactive intermediates, including neuroprotective kynurenic acid (KA) and neuroexcitatory quinolinic acid (QA). From QA, quinolinate phosphoribosyl transferase (QPRT) serves as the rate limiting enzyme in *de novo* nicotinamide adenine dinucleotide (NAD+) synthesis^15^, an essential energy source for many cellular functions^16,17^. Under inflammatory conditions, interferon (IFN)-γ, IFN-α and tumor necrosis factor (TNF)-α direct TRP metabolism toward KYN by inducing the enzyme indoleamine 2,3-dioxygenase 1 (IDO1)^18,19^. Vitamin B_6_ deficiency leads to increased 3-hydroxykynurenine (3-HK) and QA, with high 3-HK promoting oxidative stress and apoptotic neuronal death^20^ and high QA/KA ratios promoting glutamatergic excitotoxicity and further neuroinflammation^21,22^. In contrast, KA directly blocks QA excitotoxicity^23^ and protects against dopaminergic cell death^24^. KP activation has been demonstrated to occur across various inflammatory conditions, including aging^25^, multiple sclerosis^26^, Alzheimer’s disease^27–29^, schizophrenia^30^, peripartum depression^31^, lung cancer^32^, and in children with inflammatory neurological disorders^33^.

Severe KP disruption has also been reported in PD, involving increases in 3-HK, increases in the immune activation marker neopterin, and decreases in neuroprotective KA^27,34–43^. Yet, our understanding of the peripheral and central contributions to KP activation in PD remains incomplete. In addition, it is unclear whether there exists PD clinical subgroups with specific patterns of KP dysmetabolism. This study aimed to identify such endophenotypes to further support the development of individualized therapeutic strategies in PD.

## RESULTS

This multicenter study included 335 participants drawn from 8 research centers and consisted of 158 control older adult participants and 177 participants with PD. Demographic information for the study participants is shown in **Table 1** and details on the originating research centers for study population participants are presented in **Supplemental Fig. 1**. Control participants were on average 67.70 years old (SD = 7.58) while the PD participants were 67.41 years old (SD = 8.23). Consistent with disease prevalence^44^, males were overrepresented in the PD participants compared to the controls (69.4% vs. 50.6%; *P* < 0.001, Chi-Square test). There were no differences between PD and controls with respect to *APOE-ε4* carriage (23.8% vs. 24.7%; *P* > 0.05, Chi-Square test) or years of education (16.22 vs. 16.53; *P* > 0.05, two-sided Student’s *t* test). PD participants had an average time since diagnosis of 5.53 years (range = 0 to 26) and Levodopa equivalent daily dose (LEDD) of 566.15 mg (range = 0 to 2150). PD participants had lower Montreal Cognitive Assessment (MoCA) scores (24.30 ± 3.94) compared to controls (27.57 ± 2.01; *P* = 0.0002, two-sided Student’s *t* test). No differences were observed in CSF biomarkers p-tau181 or total tau (*P* > 0.05, two-sided Student’s *t* test), although CSF Aβ_42_/Aβ_40_ was slightly higher in the PD group (*P* < 0.05, two-sided Student’s *t* test).

**Figure 1:**
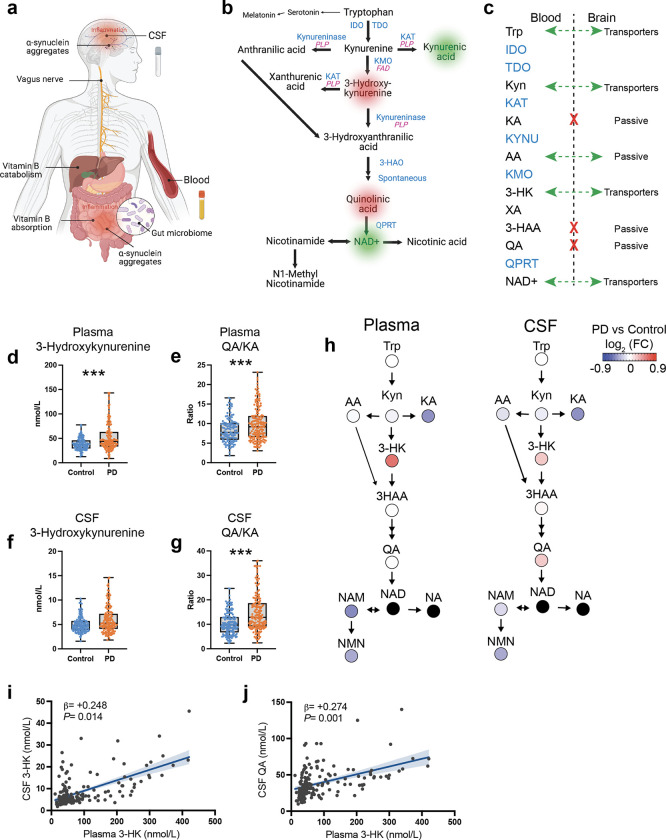
The kynurenine pathway integrates peripheral and CNS proinflammatory responses and metabolic dysfunction in Parkinson’s disease **(a)** Parkinson’s disease is a multisystem neurodegenerative disease. Recent data support that peripheral α-synuclein pathology and inflammation are related to the gut microbiome and that misfolded α-synuclein may undergo transneuronal transport via the vagus nerve to the brain. **(b)** In the kynurenine pathway, tryptophan is metabolized via IDO and TDO to kynurenine, which is converted to either 3-hydroxykynurenine by KMO, to anthranilic acid by kynureninase, or to kynurenic acid by KAT. 3-hydroxykynurenine is converted to xanthurenic acid by KAT. Kynureninase converts 3-hydroxykynurenine and anthranilic acid to 3-hydroxyanthranlic acid. 3-Hydroxyanthranilic acid is converted by 3-HAO, and further spontaneously converts to quinolinic acid, which is converted by QPRT to NAD^+^, a major energy source for cells. NAD^+^ is further converted to nicotinamide and N1-methylnicotinamide or to nicotinic acid and then to the antioxidant trigonelline. Tryptophan is also a precursor for serotonin and melatonin production. Key converting enzymes are indicated in blue. Also shown are enzymes supported by the active form of vitamin B_6_, PLP and B_2_ vitamer, FAD, in purple). Abbreviations: IDO: indoleamine 2,3-dioxygenase, 3-HAO: 3-hydroxyanthranilate-3,4-dioxygnase, FAD: flavin adenine dinucleotide, KAT: kynurenine aminotransferase, KMO: kynurenine 3-monooxygenase, PLP: pyridoxal 5’-phosphate, QPRT: quinolinic acid phosphoribosyltransferase, and TDO: tryptophan 2,3-dioxygenease. **(c)** Several kynurenine pathway metabolites (black) have high blood-brain barrier permeability (green arrows), while others are considered to cross the blood-brain barrier poorly (red X). Enzymes are shown in blue and do not cross the blood-brain barrier. High blood-brain permeability would allow passage of circulating kynurenines into brain kynurenine pools. **(d)** Box and whisker plot showing median concentration of plasma 3-hydroxykynurenine in control and PD participants. Whiskers indicate minimum to maximum data range. ***P < 0.001 analyzed by ANCOVA with age and sex included as covariates. **(e)** Ratio of plasma QA/KA in control and PD participants. ***P < 0.001 analyzed by ANCOVA with age and sex included as covariates. **(f)** Concentration of CSF 3-hydroxykynurenine in control and PD participants. Analyzed by ANCOVA with age and sex included as covariates. **(g)** CSF QA/KA in control and PD participants. ***P < 0.001 analyzed by ANCOVA with age and sex as covariates. **(h)** Metabolic pathway maps showing plasma (left) and CSF (right) kynurenine pathway metabolites. Metabolites are colored by the log2 fold-change scale comparing the PD group to the control group. Metabolites that were not measured are shown as black circles. Abbreviation FC: fold change. **(i)** Linear regression of plasma 3-HK predicting CSF 3-HK. Age and sex were included as covariates in the linear model. Shown are the β-estimates and P-values from the linear model and the 95% confidence band of the line of best fit. **(j)** Linear regression of plasma 3-HK predicting CSF QA. Age and sex were included as covariates in the linear model. Shown are the β-estimates and P-values from the linear model and the 95% confidence band of the line of best fit.

Blood and CSF KP metabolites and B-vitamin cofactors required for their enzymatic synthesis were measured using a liquid chromatography-tandem mass spectrometry (LC-MS/MS) assay with high sensitivity and precision (Fig. 1a–b). Notably, many metabolites are transported from the blood across the blood-brain barrier (BBB) by the large neural amino acid transporter^45,46^ and basal BBB permeability is susceptible to change in inflammatory disease states^47^ (Fig. 1c). Accordingly, CSF and plasma pools for many metabolites were often correlated (**Supplemental Fig. 2a-c**).

### KP activation in Parkinson’s disease

LC-MS/MS analysis revealed that plasma and CSF concentrations of the essential amino acid and KP precursor TRP did not differ between control and PD participants (*P* > 0.05, ANOCVA with age and sex included as covariates), nor did levels of KYN (*P* > 0.05; **Supplemental Fig. 3a-b**). However, differences were observed in some KP metabolites between PD participants and controls. In plasma, higher concentrations of the neuroexcitatory 3-HK were found in PD (*P* < 0.001; Fig. 1d). Similarly, PD participants had higher plasma levels of the neuroexcitatory QA/KA ratio (*P* < 0.001; Fig. 1e). Conversely, levels of neuroprotective KA were lower in PD plasma (*P* < 0.001; **Supplemental Fig. 3a**). In CSF, 3-HK was not different (*P* > 0.05; Fig. 1f), although the QA/KA ratio was higher in PD compared to controls (*P* < 0.001; Fig. 1g). CSF QA was also higher in PD compared to control (*P* < 0.05; **Supplemental Fig. 3b**). Lower concentrations of KA (*P* < 0.001) and AA (*P* < 0.05), NMN (*P* < 0.001) – an anti-inflammatory metabolite^48^ – were observed in PD. KP metabolic maps for plasma and CSF demonstrate lower plasma KA and higher 3-HK and higher CSF QA in PD (Fig. 1h). While QA is not actively transported across the BBB^27^, its substrate 3-HK is transported from the blood across the BBB by the large-neutral amino acid transporters^49^. Thus, peripheral-CNS communication through 3-HK BBB transport may contribute to the increase in neuroexcitatory CSF QA in PD. In support of this, plasma 3-HK was found to be positively associated with CSF 3-HK (β = +0.248, *P* < 0.05; Fig. 1i) and with CSF QA (β = +0.274, *P* < 0.001; Fig. 1j).

### Low B-vitamin status associates with inflammation and KP activation in Parkinson’s disease

**Figure 2:**
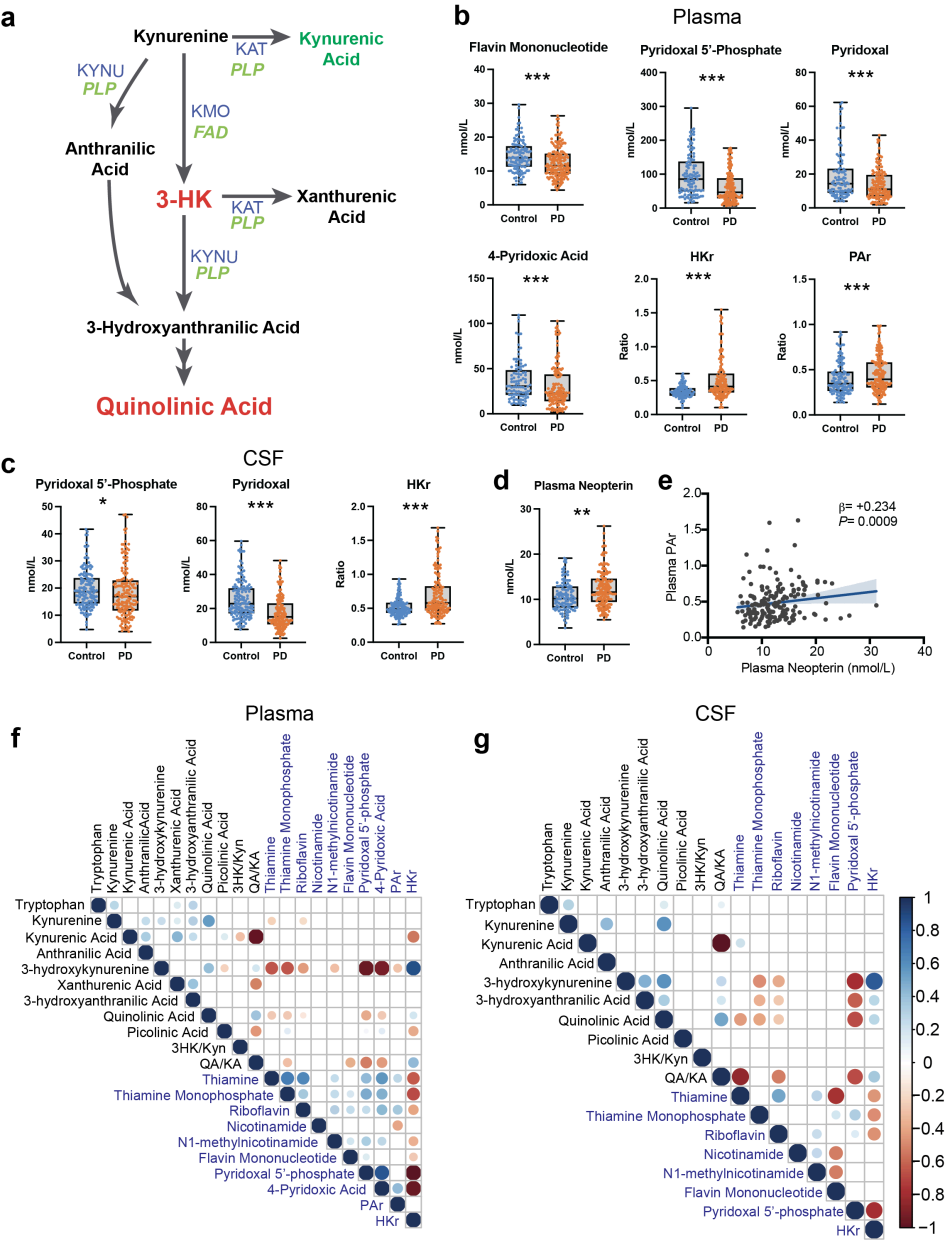
Vitamin B_6_ deficiency associates with inflammation and neuroexcitatory KP activation in PD **(a)** Kynurenine pathway enzymes (blue) require B-vitamin cofactors (green) for their activity. Abbreviations: 3-HK: 3-hydroxykynurenine, FAD: flavine adenine dinucleotide, KAT: kynurenine aminotransferase, KMO: kynurenine 3-monooxygenase, KYNU: kynureninase, PLP: pyridoxal 5’phosphate. **(b)** B-vitamins, HKr, and the PAr Index in plasma from control and PD participants. HKr is a validated functional measure of vitamin B_6_ status. PAr is an index of vitamin B_6_ catabolism. ***P < 0.001 using ANCOVA including age and sex as covariates. Abbreviations: HKr: HK ratio, PAr: PAr Index. **(c)** B-vitamins and HKr in CSF from control and PD participants. HKr is a validated functional measure of vitamin B_6_ status. *P < 0.05 and ***P < 0.001 using ANCOVA including age and sex as covariates. Abbreviations: HKr: HK ratio. **(d)** Plasma neopterin, a validated marker of inflammation, in control and PD participants. **P < 0.01 using ANCOVA including age and sex as covariates. **(e)** Linear regression reveals that plasma PAr is significantly correlated with plasma neopterin concentration. Age and sex were included as covariates in the linear model. Shown are the β-estimates and *P*-values from the linear model and the 95% confidence band of the line of best fit. PAr: PAr Index. **(f)** Correlation matrix showing association between plasma metabolites (black text) and B-vitamins (blue text) in PD participants. Age and sex were included as covariates. Significant correlations are shown with the circle size indicating the *P*-value of the correlation and color indicating size and direction of the *β*-estimate. Abbreviations: 3-HK: 3-hydroxykynurenine, HKr: HK ratio, Kyn: kynurenine, PAr: PAr Index, QA: quinolinic acid. **(g)** Correlation matrix showing association between CSF metabolites (black text) and B-vitamins (blue text) in PD participants. Age and sex were included as covariates. Significant correlations are shown with the circle size indicating the *P*-value of the correlation and color indicating size and direction of the *β*-estimate. Abbreviations: 3-HK: 3-hydroxykynurenine, HKr: HK ratio, Kyn: kynurenine, QA: quinolinic acid.

Enzymatic conversion of some KP metabolites to their subsequent products requires the active forms of vitamins B_2_ (flavine adenine dinucleotide, FAD) and B_6_ (pyridoxal 5’-phosphate; PLP) to serve as enzyme cofactors (Fig. 2a). B-vitamin levels are determined by the balance of intestinal absorption and liver catabolism to impact overall availability. The HK ratio (HKr) is a functional measure of vitamin B_6_ status, calculated as the ratio of 3-HK to the four kynurenine products of PLP-dependent enzymes KAT and KYNU (i.e., 3-HK/(KA+AA+XA+HAA))^50^. Similarly, the PAr Index is a blood-based measure of vitamin B_6_ catabolism, calculated as the ratio of plasma B_6_ catabolite 4-pyridoxic acid (4-PA) to the sum of its active form PLP and its transport form pyridoxal (PL) (4-PA/(PLP + PL)^51^ to assess vitamin B_6_ status. The PAr Index is elevated in inflammatory conditions^51,52^, cancer^53,54^, coronary artery disease^55^, stroke^56^ and depression^57^.

In examining whether B-vitamin deficiency links proinflammatory responses and KP activation in PD, it was found that plasma flavin mononucleotide (FMN, *P* < 0.001), PLP (*P* < 0.001), pyridoxal (*P* < 0.001) and 4-pyridoxic acid (4-PA, *P* < 0.001) were all lower in PD participants when compared to controls (Fig. 2b and **Supplemental Fig. 4a**). Similarly, plasma HKr (*P* < 0.001) and the PAr Index (*P* < 0.001) were higher in PD compared to control, confirming lower vitamin B_6_ status in the PD group. In CSF, PLP (*P* < 0.05) and pyridoxal (*P* < 0.001) were both lower in PD compared to control (Fig. 2c; **Supplemental Fig. 4b**) and the PD group had higher CSF HKr compared to the control group (*P* < 0.001).

Next, levels of the peripheral inflammatory marker neopterin – an indicator of monocyte/macrophage activation^58^— were assessed to further investigate the link between inflammatory responses and vitamin B_6_ catabolism. Indeed, plasma neopterin was higher in PD compared to controls (*P* < 0.01; Fig. 2d). Moreover, a positive association was demonstrated between plasma neopterin and the PAr Index (β = +0.234, *P* < 0.001; Fig. 2e). Further correlation analyses examined associations between the kynurenines and B-vitamin derivatives within plasma (Fig. 2f) and CSF (Fig. 2g) pools in the PD group. Notably, 3-HK and QA levels were higher at lower vitamin B_6_ (PLP) levels, further highlighting the possibility that vitamin B_6_ deficiency serves as a link between peripheral inflammation and the altered KP profile observed in PD.

### CSF QA associates with motor symptom severity and neurodegeneration in Parkinson’s disease

**Figure 3:**
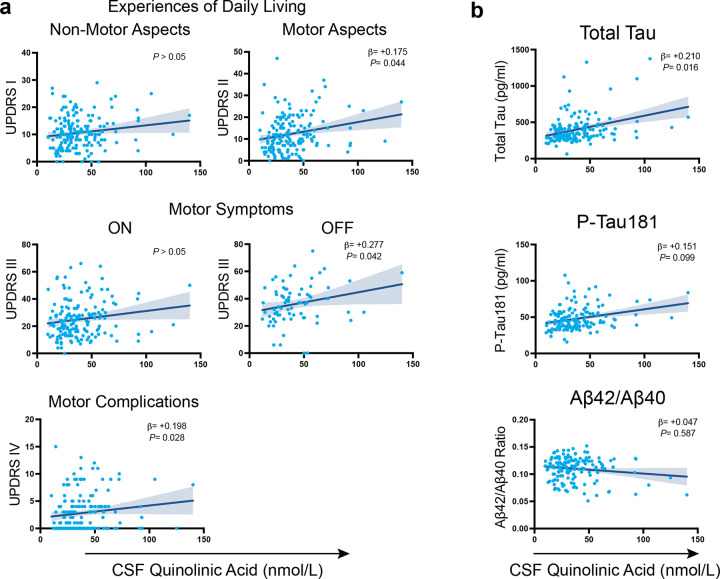
CSF QA associates with motor symptoms and tau in Parkinson’s disease **(a)** Linear regression analysis showing associations between CSF QA with MDS-UPDRS Parts I-IV scores in PD participants. Plotted is the 95% confidence band of the best-fit line from the linear regression. Age and sex were included as covariates. β-estimates and *P*-values from the linear model are shown. Abbreviation: MDS-UPDRS: Movement Disorder Society Unified Parkinson’s Disease Rating Scale, QA: quinolinic acid. **(b)** Linear regression analysis showing associations between CSF QA and core AD biomarkers CSF total tau, p-tau181, and Aβ_42_/Aβ_40_ ratio in PD participants. Plotted is the 95% confidence band of the best-fit line from the linear regression. CSF biomarkers were log-transformed in a linear model that included age and sex as covariates. β-estimates and *P*-values from the linear model are shown.

Associations between CSF QA and PD symptoms were next investigated using participant data from the Movement Disorder Society Unified Parkinson’s Disease Rating Scale (MDS-UPDRS^59^; Fig. 3a). CSF QA was positively associated with MDS-UPDRS Part II total score, which is sensitive to motor aspects of experiences of daily living (β = +0.175, *P* < 0.044), with Part III OFF, which is a measure of motor severity (β = +0.277, *P* < 0.042), and Part IV, which is related to motor complications of Levodopa replacement therapy (β = +0.198, *P* < 0.028). Additionally, associations between CSF QA with Alzheimer’s disease biomarkers CSF amyloid and tau were examined given the high comorbidity of Alzheimer’s disease neuropathology in PD^60–64^. This analysis revealed that CSF QA was associated with CSF total tau (β = +0.210, *P* < 0.016), but not CSF p-tau181 (*P* > 0.05) or CSF Aβ_42_/Aβ_40_ (*P* > 0.05; Fig. 3b), suggesting a specific link between QA-induced neurotoxicity and neurodegeneration.

### CSF and plasma KP metabolites accurately predict Parkinson’s disease

**Figure 4:**
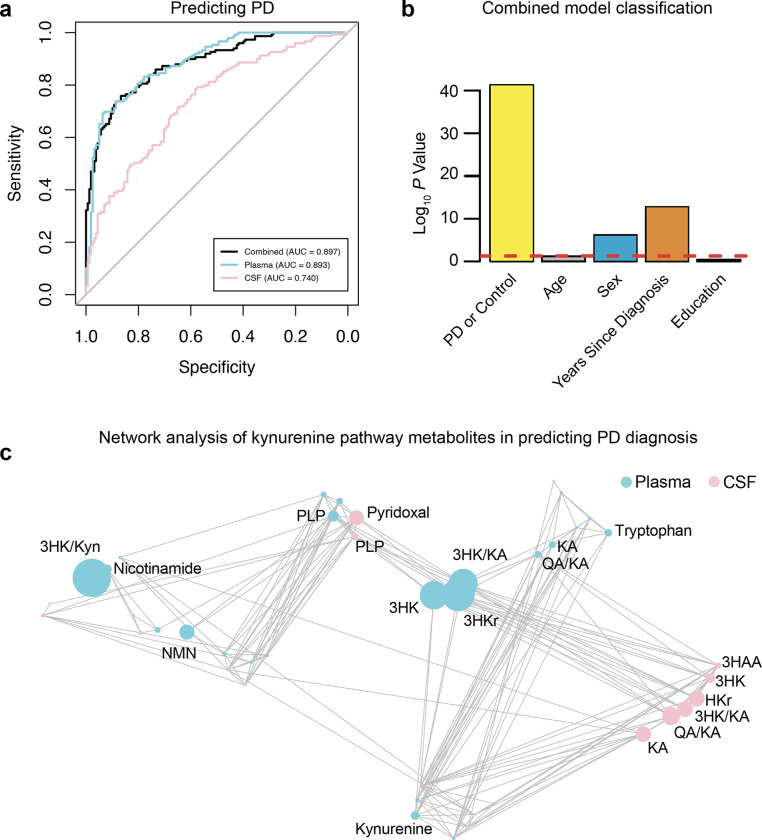
PD prediction using plasma and CSF KP metabolites and related B-vitamins. **(a)** ROC curve analysis of a combined model consisting of plasma and CSF KP-related metabolites and their ratios (black curve), and single fluid models with plasma (cyan curve) or CSF (salmon curve) in distinguishing PD participants (n = 149) from control participants (n = 158). All metabolite biomarkers were used in these models. A cross-validation strategy was employed such that half the participants were used to optimize the integrated model, after which the remaining half were presented to the model to test its performance. **(b)** Combined model performance in correctly classifying diagnosis, age, sex, years since diagnosis and education. **(c)** Network analysis revealing kynurenine pathway metabolites correlating with predicting PD vs control (Spearman correlation *P* < 0.05). Metabolites are coded according to sample type (plasma: cyan or CSF: salmon). Circle size indicates degree of association between metabolite and predicting PD diagnosis. Abbreviations: KA: kynurenic acid, 3-HK: 3-hydroxykynurenine, 3HAA: 3-hydroxyanthranilic acid, QA: quinolinic acid, NMN: N1-methylnicotinamide.

We next evaluated whether CSF and plasma kynurenines and related B-vitamin levels might be useful in distinguishing PD from controls. For this analysis all kynurenines, their ratios and related B vitamins were included in the models and a cross validation strategy was used to simultaneously optimize the integrative model on a subset of metabolite data and then test its performance on previously unseen participants. We found that both CSF (AUC: 0.740) and plasma (AUC: 0.893) metabolites correlated with PD diagnosis (AUC combined model: 0.897; Fig. 4a). Indeed, KP metabolite levels were robust predictors of PD diagnosis, sex, and years since diagnosis, but not age or years of education (Fig. 4b). Correlation-based network analysis was used to evaluate the relationships between plasma and CSF metabolite features in a bottom-up approach. In this scenario, the metabolite features are presented as nodes in the network map and links are drawn between them when a relationship is present. KP metabolite features that effectively predicted PD diagnosis included the KMO-related product/substrate ratio 3-HK/Kyn, 3-HK/KA, 3-HKr, 3-HK, PLP, QA/KA, KA, and TRP in plasma and QA/KA, 3-HK/KA – an excitotoxic ratio^36^ – along with HKr, KA, PL, PLP, 3-HK and 3-HAA in CSF (Fig. 4c). Together, these results demonstrate the utility of the KP, particularly in plasma, to serve as a reliable biofluid biomarker for distinguishing PD from controls.

### Parkinson’s disease clinical subgroups display distinct patterns of KP alterations and distinct clinical features

**Figure 5:**
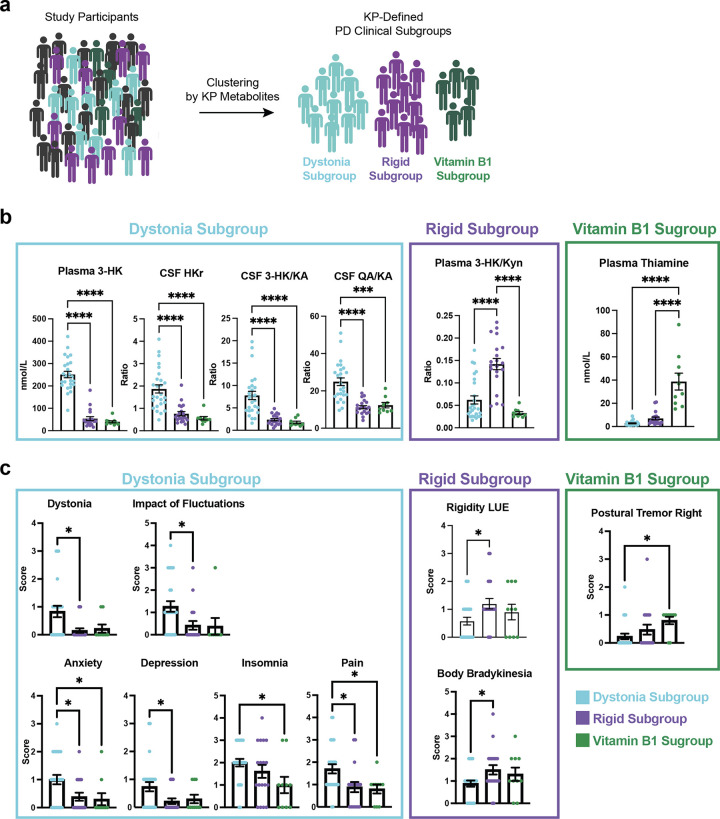
Parkinson’s disease clinical subgroups show distinct clinical endophenotypes. **(a)** Schematic depicting study participant subgrouping by KP metabolite data. **(b)** Kynurenine pathway metabolite and B-vitamin markers of the PD clinical subgroups. Increased KP metabolites marking the Dystonia Subgroup included plasma 3-HK, CSF HKr, CSF 3-HK/KA and CSF QA/KA. The Rigid Subgroup was marked by increased levels of plasma 3-HK/Kyn. The Vitamin B1 Subgroup was marked by high plasma thiamine levels found to be in the range of vitamin supplementation. Group differences were assessed using one-way ANOVA with Tukey’s post hoc test for pairwise comparisons. ***P < 0.001 and ****P < 0.0001. **(c)** Clinical features associating with each of the PD clinical subgroups. The Dystonia Subgroup had higher MDS-UPDRS scores on Dystonia and Impact of Fluctuations compared to the other subgroups. In addition, the Dystonia Subgroup had higher scores for Anxiety, Depression, Insomnia, and Pain. Conversely, the Rigidity Subgroup had lower scores across these domains but did have higher Rigidity LUE and Body Bradykinesia. Group differences were assessed using one-way ANOVA with Tukey’s post hoc test for pairwise comparisons. *P < 0.05. Abbreviations: LUE: left upper extremity.

Characterizing the clinical and biochemical heterogeneity of Parkinson’s disease is critical to understanding its origins and devising targeted management strategies^3^. Therefore, given the size of our multi-center patient cohort, we applied unbiased t-Sne clustering to better define the heterogeneity of our study population using metabolite data (Fig. 5a, **Supplemental Fig. 5a**). Note that clinical features were not included in the clustering at this stage. Thus, by using metabolite data alone, we preliminarily identified three small yet distinct PD clinical subgroups with specific KP metabolic profiles which clustered separately from the majority of other PD patients and all controls. Further exploratory analyses were undertaken to better define their distinguishing features, and we were able to define these subgroups as: 1) the Dystonia Subgroup (n=25); 2) the Rigid Subgroup (n=20); and 3) the Vitamin B1 Subgroup (n=10; Fig. 5b). These PD clinical subgroups did not show differences in years since diagnosis or age (**Supplementary Fig. 5b,c**), suggesting they represent separate pathophysiologic trajectories as opposed to distinct stages along the same trajectory. PD participants in the Dystonia Subgroup had high plasma 3-HK, CSF HKr, CSF 3-HK/KA and CSF QA/KA ratio in comparison to PD participants in the other subgroups. In contrast, the Rigid Subgroup had high plasma 3-HK/Kyn ratio without correspondingly high QA. The Vitamin B1 Subgroup had higher levels of plasma thiamine and thiamine monophosphate (TMP; **Supplemental Fig. 5d**).

MDS-UPDRS clinical scores across subgroups were assessed to determine whether the identified PD clinical subgroups were related to specific clinical dysfunction (Fig. 5c). Indeed, the Dystonia Subgroup had more severe dystonia and were more likely to report a functional impact of motor fluctuations on their daily activities and social interactions compared to the other subgroups. In terms of non-motor symptoms, they also reported greater anxiety, depression, insomnia, and more painful OFF-state dystonia. In contrast, the Rigid Subgroup reported higher rigidity in the left upper extremities and higher body bradykinesia. The Rigid Subgroup also had a lower mean LEDD compared to the other PD subgroups (**Supplemental Fig. 5e**). In terms of symptoms, the Vitamin B1 Subgroup reported greater right-sided postural tremor in comparison to the other PD clinical subgroups.

## DISCUSSION

The present study revealed KP dysregulation in PD is linked to peripheral and CNS inflammation and low vitamin B_6_ status. The shift in KYN metabolism was characterized by lower neuroprotective KA and higher 3-HK and QA, which are both neuroexcitatory compounds. KP metabolic maps for plasma and CSF showed that lower KA and higher 3-HK were observed in both plasma and CSF pools in PD, however, higher QA was only observed in CSF. This observation suggests BBB permeable 3-HK may serve as a conduit for CNS-periphery crosstalk, whereby peripheral 3-HK traffics to increase CNS 3-HK pools and ultimately increase neuroexcitatory QA. While future studies should aim to provide experimental evidence of the BBB transfer of 3-HK directly, we found that 3-HK concentrations in CSF and plasma were significantly correlated. Amid high CSF QA we observed more severe PD motor symptoms and higher CSF tau. An exploratory analysis of PD patient subgroups suggests the possibility that specific alterations in KP metabolism may be linked to distinct clinical dysfunction.

Results of the current study suggest KP dysfunction might result from vitamin B_6_ deficiency shifting production away from the neuroprotective KA and AA and towards overproduction of neuroexcitatory 3-HK and QA. As a key cofactor, vitamin B_6_ is required for the enzymatic activities of KYNU and KAT^65^, which produce AA and KA, respectively. Consistent with previous work^52^, the current study demonstrated that the plasma marker of inflammation neopterin was linked to B_6_ deficiency, directly implicating peripheral inflammation in low vitamin B_6_ status. The proposed connection between B_6_ deficiency, KP dysmetabolism, and consequent neuroinflammation may further explain epidemiological studies showing reduced dietary B6 intake is tied to increased PD risk^66^. Vitamin B6 catabolism (and consequently PAr) rises in inflammatory conditions^51^ and peripheral inflammation may both drive or potentiate PD-associated B_6_ deficiency. B_6_ deficiency might also be related to levodopa administration, as previous work has shown that individuals taking more than 2,000 mg LEDD were nearly always found to have B_6_ deficiency^67^. In this case, B_6_ deficiency may be explained by consumption of PLP during levodopa metabolism and deactivation of PLP by carbidopa^68–71^. Thus, additional work is required to determine the source of B_6_ deficiency in PD and data on intake of B_6_ and the other vitamins would be useful.

The current study also supports the KP may be capable of distinguishing PD clinical subgroups with distinct pathophysiology and clinical profiles. While these findings should be validated in larger studies, interesting preliminary associations were observed. For example, the Dystonia Subgroup was biologically defined by high levels of neuroexcitatory and inflammatory CSF QA/KA. This group also showed greater involuntary muscle contractions (dystonia) and reported greater impact of their fluctuations. The Dystonia Subgroup also showed greater anxiety, depression and pain compared to the other subgroups. Interestingly, these symptoms may be consistent with the “Anxious fluctuators” clinical endophenotype previously identified using only clinical variables^72^. This clinical subgroup was uncovered in the current study using only metabolite data, potentially providing a biological substrate for this previously identified clinical endophenotype. The KP alterations specific to the Dystonia Subgroup were increased neuroexcitatory and neuroinflammatory CSF QA/KA. In contrast, the Rigid Subgroup showed higher rigidity and body bradykinesia. Together with akinesia, these are symptoms commonly defined as motor deficits relating to the akinetic/rigid (AR) type of PD. AR symptoms have been linked to gray matter decline and altered functional connectivity within frontal-parietal networks critical for motor planning and execution^73^, and as such, the AR motor subtype is associated with poorer prognosis and increased risk of dementia compared to the tremor subtype^74,75^. The Rigid Subgroup did not show increased CSF QA/KA, but rather increased 3-HK/Kyn, which may indicate elevated KMO activity. That differences in clinical symptoms correspond to differences in KP profiles, with increased CSF QA/KA and normal 3-HK/Kyn in the Dystonia Subgroup, and normal CSF QA/KA but abnormal 3-HK/Kyn in the Rigid Subgroup, suggests the KP may be sensitive to specific PD endophenotypes.

Distinct KP alterations may provide insight into the clinical trajectory associated with each symptom dimension/profile and inform person-tailored therapeutic strategies. For example, PD patients with altered KP metabolism may benefit from strategies aiming to restore vitamin B_6_ levels and reducing peripheral inflammation. Indeed, KMO inhibition has been tested as therapeutic strategy for Alzheimer’s and PD, although these drugs are complicated by low BBB permeability. However, recent second generation KMO inhibitors with increased BBB permeability have shown promise in reducing the production of neuroexcitatory KP metabolites and in shifting the pathway towards neuroprotective KA^76^. An alternative strategy to address KP dysfunction might involve exercise, which can increase KAT expression by directing peripheral KYN metabolism toward KA production^77^. Combinational therapies incorporating anti-inflammatory agents, KMO inhibitors or KAT enhancers, in addition to lifestyle factors such as exercise, may have synergistic effects.

This study has several strengths, including its multicenter design, analysis of kynurenines and B-vitamins in matched CSF and plasma pools in the same sample, and application of unbiased machine learning approaches. Several opportunities to extend on our findings remain open. First, because of the cross-sectional design, longitudinal studies should be undertaken to examine the changes in KYN metabolism over the course of disease development. For example, it is possible that metabolites fluctuate within different clinical populations or across an individual person’s disease trajectory, and longitudinal study may reveal coupling between metabolites and clinical dysfunction. Second, in keeping with PD prevalence, our study had an overrepresentation of males in the PD cohort, which makes it difficult to assess potential sex differences. Importantly, previous studies have shown sex-specific differences in the KP^78,79^. Third, given that this is an observational study, it is difficult to determine whether the observed KP disruptions are a cause or consequence of PD pathophysiology. Interventional studies and preclinical experiments will be essential in determining causality. Fourth, PD diagnosis was made based on patient-reported history and neurological exam using the United Kingdom Parkinson’s Disease Society Brain Bank diagnostic criteria, but still may not be fully accurate^80^. Synuclein seeding assays would be useful to confirm synucleinopathy across the cohort^81^. Fifth, the PD clinical subgroup populations were of modest sample size and these findings should be expanded, ideally in a large cohort of *de novo* PD participants. Nevertheless, that these subgroups emerged in the current multicenter design lends support to the external validity of the subgroup findings. Finally, information on dietary intake of B-vitamins or use of vitamin supplements was not available for all participants. This information would be useful in future studies aimed at directly assessing relationships between vitamin status and KP activation in PD participants.

In conclusion, the current study identifies the KP as a site of peripheral-cerebral crosstalk, integrating inflammation and metabolic dysfunction. Furthermore, this study provides new evidence to suggest that the KP can reflect specific PD endophenotypes. Identification of PD clinical subgroups widens opportunities for developing biomarker candidates, novel avenues for investigating disease pathogenesis and new strategies for person-tailored therapeutic targeting.

## MATERIALS AND METHODS

### Study participants and study design

Three-hundred and twenty-five participants were included in this multicenter study and were drawn from 8 research centers (**Supplementary Fig. 1**). This included participants from 1) the Stanford Movement Disorders Clinic (Stanford MDC); 2) the Stanford Alzheimer’s Disease Research Center (Stanford ADRC); 3) the Stanford Aging and Memory Study (SAMS); 4) the VA Puget Sound Health Care System/University of Washington (Seattle VA); 5) the VA Portland Medical Center (Portland VA); 6) the Oregon Health & Science University Layton Aging and Alzheimer’s Disease Research Center (OHSU ADRC); 7) the University of Pennsylvania Alzheimer’s Disease Center (Penn ADC); and 8) the University of Pennsylvania Frontal Temporal Dementia Center (Penn FTDC). Participants from the Stanford MDC and Stanford ADRC were included if they were cognitively normal healthy adults or if they met United Kingdom Parkinson’s Disease Society Brain Bank clinical diagnostic criteria for PD. Healthy control participants were recruited from the family of PD participants and from the surrounding community. They had no history of PD, other neurodegenerative diseases, or chronic neuropsychiatric disorders. SAMS participants were clinically unimpaired older adults with Clinical Dementia Rating global score of zero^82^. A full neuropsychological battery assessing multiple domains was given to assign a cognitive diagnosis. Participants from Stanford were defined as cognitively impaired if scores were ≥ 1.5 standard deviations below age- and education-matched normative values on at least two separate neuropsychological measures, regardless of domain^83^. Seattle VA and Portland VA and OHSU ADRC assigned diagnoses at a clinical consensus conference. Control participants from Penn ADC and Penn FTDC underwent cognitive testing and determination of cognitive diagnosis by clinical consensus after the time of sampling. Levodopa equivalent daily dose (LEDD) was determined as previously described^84^. Study protocols were approved by Institutional Review Boards at each center. In accordance with the Declaration of Helsinki, written informed consent was obtained from each study participant or their legally authorized representative.

## MDS-UPDRS

The Movement Disorder’s Society Unified Parkinson’s Disease Rating Scale (MDS-UPDRS) was used to evaluate various aspects of Parkinson’s disease including non-motor and motor experiences of daily living and motor complications^59^.

### Plasma and CSF collection

For the Stanford MDC, Stanford ADRC and SAMS, fasting plasma was collected within 2 weeks of lumbar puncture. For the remaining cohorts, CSF and plasma blood draws occurred on the same morning. No hemolysis or discoloration was apparent in any of the included CSF or plasma samples (visual inspection). CSF samples were restricted to two freeze-thaw cycles, as recommended by Consensus of the Task Force on Biological Markers in Psychiatry of the World Federation of Societies of Biological Psychiatry^85^.

### Liquid chromatography-tandem mass spectrometry

Liquid chromatography-tandem mass spectrometry (LC-MS/MS) was performed on CSF and plasma samples by Bevital AS (Bergen, Norway, www.bevital.no)) and measured kynurenine pathway metabolites and related vitamins including tryptophan, kynurenine, kynurenic acid, quinolinic acid, picolinic acid, 3-hydroxykynurenine, anthranilic acid, 3-hydroxanthranillic acid, nicotinamide, N^1^-methylnicotinamide, pyridoxal, pyridoxal 5’-phosphate, thiamine, riboflavin, flavin mononucleotide, thiamine monophosphate, and neopterin, as previously described ^86^.

### Measurement of AD CSF core biomarkers

CSF amyloid and tau were measured by the Stanford ADRC Biomarker Core using the fully automated Lumipulse *G* assays (Fujirebio Diagnostics, US, Malvern, PA) on the Lumipulse G1200 instrument as previously described^12,87^. Investigators were blind to clinical and demographic information of the sample.

### Predictive modeling and subgroup analysis

A flexible machine learning method was applied to predict whether CSF and plasma levels of kynurenine pathway metabolites and related B-vitamins might be used to distinguish PD from control individuals. All participants having both plasma and CSF data were included in machine learning analysis, resulting in cohort of PD participants (n = 149) from control participants (n = 158). Metabolites and ratios included were thiamine, thiamine monophosphate, flavin mononucleotide, riboflavin, nicotinamide, N1 methylnicotinamide, pyridoxal 5’-phosphate, pyridoxal, tryptophan, kynurenine, kynurenic acid, anthranilic acid, 3-hydroxykynurenine, 3-hydroxyanthranilic acid, quinolinic acid, picolinic acid, 3-HK/KA, HKr, QA/KA in CSF and thiamine, thiamine monophosphate, flavin mononucleotide, riboflavin, nicotinamide, N1-methylnicotinamide, pyridoxal 5’-phosphate, pyridoxal, 4-pyridoxic acid, tryptophan, kynurenine, kynurenic acid, anthranilic acid, 3-hydroxykynurenine, xanthurenic acid, 3-hydroxyanthranilic acid, quinolinic acid, picolinic acid, 3-HK/KA, HKr, QA/KA, Par, and 3-HK/Kyn in plasma. The outcome of interest was PD vs control. Specifically, a two-step procedure was used to train the prediction model. The first step involved building two prediction models using random forest with metabolic features from CSF and plasma as input and the second step combined the predictions from two models built in the first step to generate a final prediction. Prediction models from the first step were also kept for comparison purpose. A cross validation strategy was used to simultaneously build the model on a subset of metabolite data and then to test its performance on previously unseen participants as previously described^88^. The model performance in cross-validation was measured via area under the ROC curve (AUC). Also tested was whether kynurenine pathway metabolite levels were robust predictors of sex, years since diagnosis, age or years of education. Correlation network analysis was used to evaluate the relationships between plasma and CSF metabolite features in a bottom-up approach. Separately, unsupervised dimension-reduction using t-distributed stochastic neighbor embedding (tSNE) algorithm^89^ was performed on all metabolites to project the individual participants into a two-dimensional plane for potential pattern detection. Based on tSNE projection, multiple PD participant clusters were identified.

## Statistical analysis

Differences in demographic variables between PD and control participants were assessed using Student’s t test or Chi-square test. CSF and plasma biomarkers were log-transformed to approximate Gaussian distribution. To evaluate differences between PD and control diagnostic groups, data were analyzed using analysis of covariance (ANCOVA) adjusting for age and sex. Similarly, multiple linear regression analyses were used to assess associations between continuous biomarker variables also after adjusting for age and sex. ANOVA was used to assess differences in selected variables between identified PD participant clusters. For metabolite data, data points below first quartile minus 1.5*IQR, or above third quartile plus 1.5*IQR were identified as outliers and removed from analysis. Outliers were not excluded from the subgroup analysis. Statistical analyses were performed using R, version 3.6.1 (R Foundation for Statistical Computing) and GraphPad Prism, version 10 (GraphPad Software, Boston, MA) and figures were prepared using BioRender (Toronto, Ontario, Canada). All statistical tests were two-sided and significance was defined as *P* < 0.05.

## Data Availability

Anonymized data are available to qualified researchers upon request to the corresponding author or by contacting the individual cohort studies.
